# Volumetric 3D Printing and Melt‐Electrowriting to Fabricate Implantable Reinforced Cardiac Tissue Patches

**DOI:** 10.1002/adma.202504765

**Published:** 2025-08-05

**Authors:** Lewis S. Jones, Hector Rodriguez Cetina Biefer, Manuel Mekkattu, Quinten Thijssen, Alessio Amicone, Anna Bock, Miriam Weisskopf, Dennis Zorndt, Debora Meier, Li Zheng, Melanie Generali, Robert K. Katzschmann, Omer Dzemali

**Affiliations:** ^1^ Soft Robotics Laboratory ETH Zurich Tannenstrasse 3 Zurich 8092 Switzerland; ^2^ Department of Cardiac Surgery University Hospital Zurich Rämistrasse 100 Zurich 8091 Switzerland; ^3^ Department of Cardiac Surgery City Hospital Zurich—Triemli Center for Experimental and Translational Cardiology (CTEC) University of Zurich Zurich 8952 Switzerland; ^4^ Polymer Chemistry and Biomaterials Group Centre of Macromolecular Chemistry Department of Organic and Macromolecular Chemistry Ghent University Krijgslaan 281 S4 Ghent 9000 Belgium; ^5^ Institute for Biomechanics ETH Zurich Gloriastrasse 37–39 Zurich 8092 Switzerland; ^6^ Center for Preclinical Development University Hospital Zurich University of Zurich Strickhofstrasse 40a Zürich 8057 Switzerland; ^7^ Institute for Regenerative Medicine (IREM) University of Zurich Schlieren 8952 Switzerland; ^8^ Mechanics & Materials Lab Department of Mechanical and Process Engineering ETH Zürich Leonhardstrasse 21 Zürich 8092 Switzerland

**Keywords:** biofabrication, cardiac regeneration, cardiac patches, cardiac tissue engineering, cardiomyocytes, implants, melt‐electrowriting, volumetric 3D printing

## Abstract

Cardiac patches to repair myocardial defects require mechanically stable materials that prevent bleeding and can be implanted via suturing. The current clinical standard, bovine pericardial patches (BPPs), serve this purpose but do not degrade or integrate with the myocardium, limiting their long‐term effectiveness. Here, we present the reinforced cardiac tissue patch (RCPatch). This multimaterial patch comprises a stiffness‐tuned, cardiomyocyte‐infiltrated 3D metamaterial and a suturable, hydrogel‐infiltrated mesh to reduce permeability and bleeding. Anisotropic metamaterials are designed and computationally optimized using a generative modeling approach and fabricated from poly(ε‐caprolactone) (PCL) via volumetric 3D printing (VP). The metamaterial supports the infiltration of cardiomyocytes, which are viable and contract in vitro. The implantability and low blood permeability of the patch is enabled by adding a melt‐electrowritten (MEW) mesh infiltrated with a fibrin hydrogel. In an acute large animal trial, the RCPatch was applied on an induced myocardial defect, where it withstood intraventricular blood pressure, prevented bleeding, and enabled hemodynamic restabilization (intraventricular pressure of 81 mmHg before, vs 66 mmHg after implantation). These findings establish a scalable framework for fabricating cardiac tissue patches that integrate mechanical reinforcement with biological function, offering a surgically implantable and future regenerative solution for intraventricular myocardial repair.

## Graphical abstract

This study presents an implantable reinforced cardiac patch (RCPatch) combining volumetrically 3D‐printed metamaterials with melt‐electrowritten (MEW) meshes. The design integrates tunable stiff polymer structures with soft, cell‐laden hydrogels. The RCPatch withstands suturing, intraventricular pressure, and cardiac contraction in a large animal myocardial defect model. The patch prevents bleeding and enables partial hemodynamic restabilization, demonstrating its potential for myocardial repair.

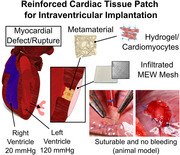

## 1 Introduction

Heart disease is the leading cause of death worldwide.^[^ ^1^, ^2^ ^]^ Myocardial Infarction (MI) occurs when blood flow to the heart is restricted, causing cardiomyocyte death, scar tissue formation, and myocardial remodeling. These changes reduce the heart's efficiency, increasing the mechanical load on surrounding tissue and causing the infarcted region to thin.^[^ ^3^ ^]^ In severe cases, this leads to myocardial rupture, which requires immediate surgical intervention.^[^ ^4^, ^5^, ^6^ ^]^ Here, cardiac patches made from biological (bovine pericardium), synthetic materials (polytetrafluoroethylene (PTFE), or Dacron (polyester fiber)) are implanted to stabilize the heart.^[^ ^7^ ^]^ However, these materials do not degrade, contract, or integrate into the myocardium.^[^ ^8^ ^]^ Furthermore, these patches undergo undesirable biological interactions such as calcification, thrombosis, and inflammation.^[^ ^9^, ^10^, ^11^, ^12^, ^13^ ^]^ These drawbacks hinder the application of cardiac patches in pediatric patients, impairing long‐term recovery and safety in many cases.^[^ ^11^, ^14^, ^15^ ^]^

An ideal cardiac patch would be implantable, easy to handle surgically and provide short‐term mechanical support while promoting biological regeneration of the damaged myocardium. Such a patch would fully integrate with native tissue, degrade in a controlled manner, and avoid triggering an immune response or other adverse effect. Tissue‐engineered cardiac patches, or engineered heart tissues (EHTs), offer a potential solution to these challenges.^[^ ^8^, ^16^ ^]^ Previous research has shown that large, clinically relevant cardiac tissues can be fabricated^[^ ^17^ ^]^ and engrafted onto animal hearts, where they maintain their structural and electrical properties,^[^ ^18^, ^19^, ^20^ ^]^ undergo vascularization, and improve cardiac function.^[^ ^21^, ^22^, ^23^, ^24^, ^25^, ^26^ ^]^ However, tissue‐engineered cardiac patches are primarily applied to the epicardial surface of the heart,^[^ ^27^, ^28^, ^29^, ^30^ ^]^ and few examples of intraventricular implantation exist.^[^ ^21^ ^]^ This is due to the low mechanical strength and limited suture retainability of current tissue‐engineered patches.^[^ ^8^, ^20^, ^31^, ^32^, ^33^, ^34^ ^]^

In this work, we developed an implantable, intraventricular cardiac patch by reinforcing EHTs with 3D‐printed polycaprolactone (PCL) materials. A key challenge in designing intraventricular cardiac patches is balancing the biological compatibility of soft materials with the mechanical robustness required for implantation. Hydrogels lack the structural integrity to withstand surgical handling and myocardial forces, while stiff synthetic materials do not support cellular integration. To address this, we utilized volumetric 3D printing (VP)^[^ ^35^, ^36^ ^]^ to fabricate a porous PCL metamaterial that could be infiltrated with a cell‐laden hydrogel and provide tunable mechanical properties that match the myocardium. We combined our metamaterial with a hydrogel‐infiltrated melt‐electrowritten (MEW) mesh, which reduces permeability and enables patch implantation via suturing. This multi‐material design enabled the RCPatch to be implanted in an acute large animal trial, where it withstood intraventricular pressure, prevented bleeding, and enabled hemodynamic restabilization (partial restoration of blood pressure and heart rate), demonstrating its potential for myocardial defect repair.

This work advances the state of the art in three key ways: i) Moving from 2D to 3D cardiac patches by developing a 3D cardiac patch for intraventricular applications; ii) Integrating tunable stiff materials with soft, cell‐laden hydrogels to create a mechanically robust cardiac tissue patch; iii) Showing that hydrogel infiltrated MEW meshes are suturable and reduce permeability, thereby preventing bleeding upon implantation.

## 2 Results

### 2.1 Design of an Intraventricular Cardiac Patch

**Figure 1:**
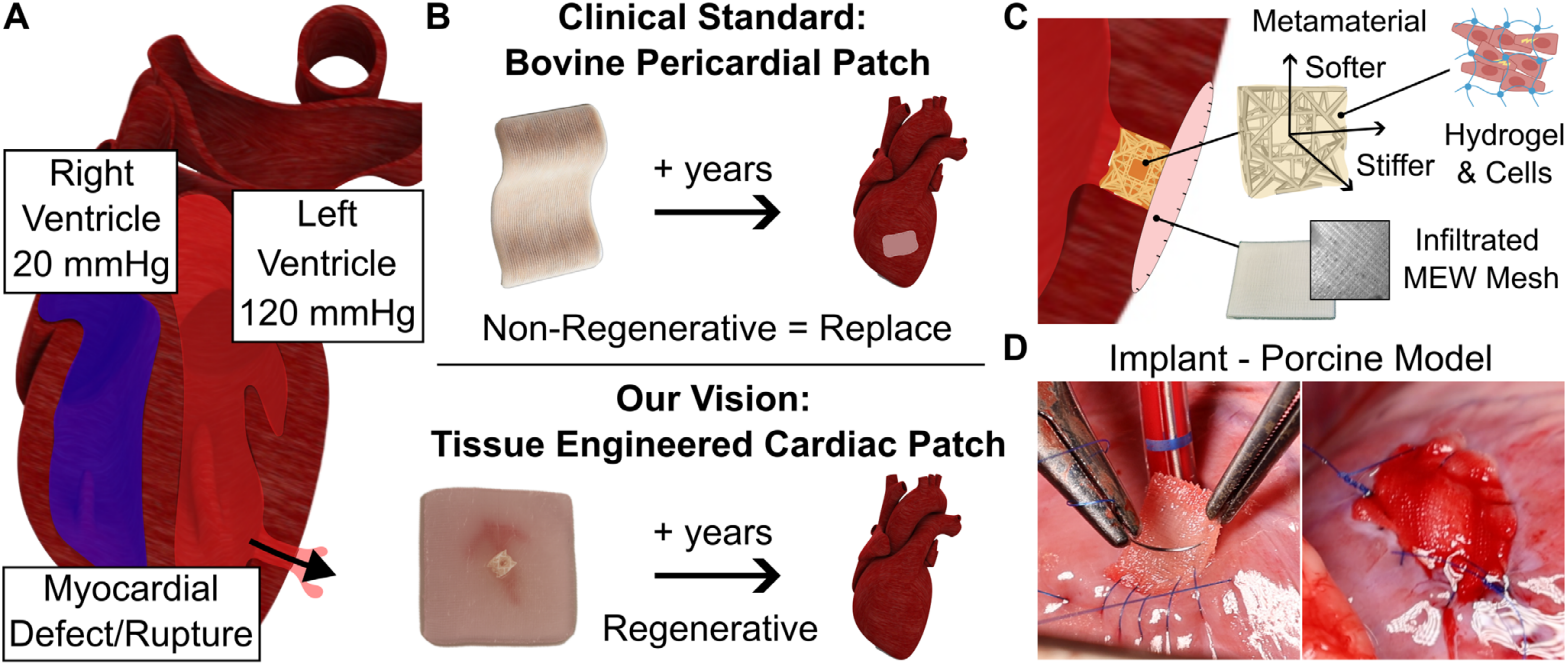
Overview of the Reinforced Cardiac Tissue Patch. A) Schematic showing a myocardial defect. Such defects cause bleeding from the left ventricle into the pericardium (ventricle rupture), or the right ventricle (septal rupture). B) The bovine pericardial patch (BPPs) compared to the reinforced cardiac tissue patch (RCPatch). BPPs remain permanent, non‐resorbable implants, which can lead to various adverse effects. Our vision with the RCPatch is long‐term integration and regeneration of the myocardium. C) RCPatch overview. The RCPatch was fabricated by infiltrating a 3D‐printed scaffold with cardiomyocytes. The scaffold comprises a metamaterial with myocardium‐like material properties, and melt‐electrowritten (MEW) mesh for implantability. D) Overview of RCPatch implantation into an animal model with an induced myocardial defect (an Ø8 mm hole through the left ventricle). The images show suturing (left) and complete defect closure (right).

We aim to improve the current clinical standard (BPPs) by repairing myocardial defects using biodegradable and regenerative cardiac patches (**Figure** **1**).^[^ ^11^ ^]^ Our patch, therefore, must mechanically stabilize the myocardial defect, withstand intraventricular blood pressure, and provide a source of cardiomyocytes. Designing such a cardiac patch is challenging due to the contraction of the heart and dynamic blood pressure (20–120 mmHg). Additionally, the patch must withstand tensile forces during suturing and implantation. The patch should also encourage favorable biological processes (e.g., cellular infiltration, viability, adhesion, etc.) while resisting unfavorable ones (e.g., calcification, inflammation, hemolysis, etc.) (see Table S1, Supporting Information for a complete list of functional requirements).^[^ ^8^, ^37^ ^]^

In our proof‐of‐concept study, we prioritized engineering a patch that could be implanted to restore hemodynamic stability, with the potential to be biodegradable and regenerative (Figure 1A,B).^[^ ^38^ ^]^ To achieve this, our RCPatch comprises three main components (Figure 1C): a MEW mesh that obstructs blood flow and allows for suturing (Figure 1D), a VP metamaterial for cell infiltration and myocardium‐like properties, and induced pluripotent stem cell cardiomyocytes (iPSC‐CMs) to support cardiac recovery.

### 2.2 Design and Fabrication of Metamaterials for Cardiac Tissue Engineering

Our cardiac patch uses a 3D‐printed metamaterial to reinforce the EHT. Truss‐based metamaterials were selected for their tunable and porous structure, making them suited for tissue engineering.^[^ ^39^ ^]^ The myocardium exhibits dynamic mechanical properties that vary during the contraction cycle (systole and diastole) and with the progression of heart disease. Consequently, the reported tissue stiffness ranges from 20 to 500 kPa, with anisotropic and auxetic characteristics.^[^ ^40^, ^41^, ^42^ ^]^ Therefore, using metamaterials provides a highly adaptable framework, enabling the tuning of material properties to match the specific mechanical demands of the application.

**Figure 2:**
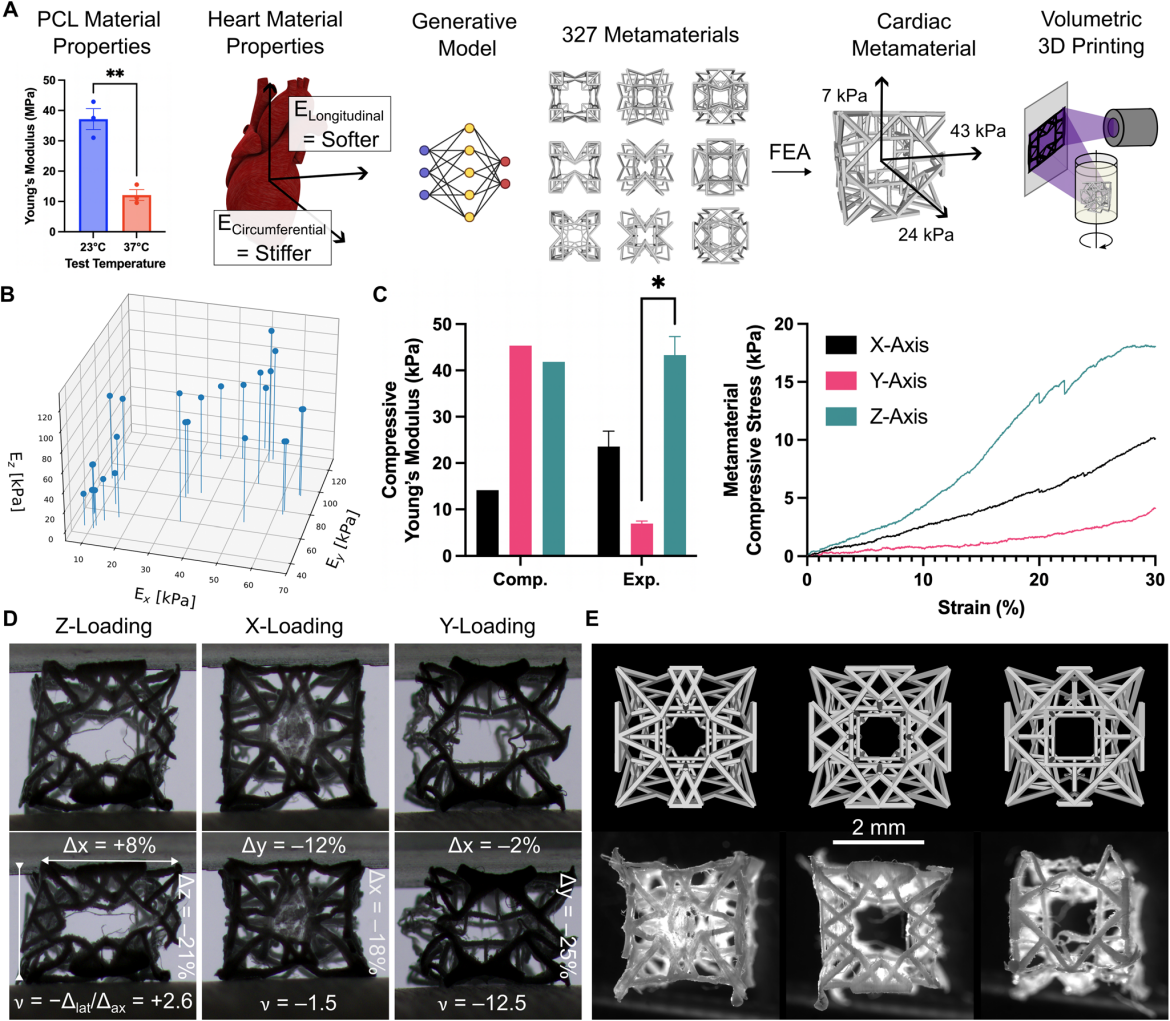
Design, Fabrication, and Characterization of Cardiac Metamaterials. A) Cardiac metamaterial design and fabrication. VP‐PCL (volumetrically 3D printed‐PCL) material properties were obtained at 37 °C (*n* = 3, unpaired *t*‐test) and used with the myocardium stiffnesses in a generative model to design metamaterial structures. The stiffnesses of the resulting structures were computed with FEA. The metamaterials were then printed with VP. B) Computed material properties of selected metamaterials under compression. C) Left: Computational versus experimental material properties for a selected metamaterial (*n* = 3, one‐way ANOVA, ^*^ *p* < 0.05). Right: experimental stress‐strain curve of the selected metamaterial. D) Photos showing selected metamaterial under compressive load. Annotations show the change in length and width and calculated Poisson ratio (ν) under compression (≈ 20%). E) Comparison between the metamaterial model (top) and printed metamaterial (bottom).

We evaluated several 3D printing techniques to fabricate high‐resolution (≈ 200 µm beam diameter) truss‐based metamaterials (Table S2 and Figure S1, Supporting Information). Ultimately, we decided to use volumetric 3D printing using photo‐cross‐linkable PCL (VP‐PCL) (**Figure** **2**). This method has previously been used to fabricate structures at high resolution (≈ 100 µm beam diameter).^[^ ^35^, ^36^ ^]^ Furthermore, PCL has various favorable mechanical (e.g., toughness), chemical (e.g., biodegradability), and biological properties (e.g., biocompatibility), for our application.^[^ ^21^ ^]^

To fabricate metamaterials using VP‐PCL, we optimized the printing ink and parameters (Table S3, Supporting Information). More specifically, we made modifications to enable the printing of larger scaffolds. During printing, light attenuation (by the photoinitiator) must be compensated to enable consistent polymerization throughout the structure. We iterated through several photoink formulations to balance reactivity, inhibition, and attenuation (TPO:TEMPO ratio and concentration). Furthermore, we optimized printing parameters, such as dose, to  fabricate scaffolds without overpolymerization (Figure S2, Supporting Information). We additionally optimized the procedure to post‐process and isolate the scaffolds (Text S2, Supporting Information).

We created a library of anisotropic truss‐metamaterials with the desired directional Young's moduli (*E_x_*,*E_y_*, and *E_z_*) by using a graph‐based deep‐learning framework.^[^ ^39^ ^]^ To do this, we first measured the material properties of VP‐PCL at body temperature (12.1 ± 1.8 MPa at 37 °C, versus 37.2 ± 3.4 MPa at room temperature) (Figure 2A; Figure S3, Supporting Information). We then used these properties, alongside the target properties of the native myocardium (one softer axis (E_Longitudinal_ ≈ 20 kPa), two stiffer axes (E_Circumferential_ ≈ 100 kPa) and auxetic) to design our truss‐metamaterials. This process yielded 327 metamaterials with similar mechanical behavior. These metamaterials were first evaluated using a Finite Element Analysis (FEA) model, and then according to manufacturability, to select the optimal design. The additional FE modeling was necessary due to the deviation between the simulation setup in the generative modeling framework (based on FE homogenization with periodic boundary conditions) and our single‐unit cell metamaterial structure.^[^ ^39^, ^43^ ^]^

The FEA validation process yielded a sub‐library of 26 metamaterials (Figure 2B), with directional Young's moduli ranging from 9–127 kPa (X‐axis (9‐66 kPa), Y‐axis (38‐124 kPa), Z‐axis (32‐127 kPa)). The other truss designs were excluded due to insufficient contraction capabilities or issues encountered during the FEA simulations, such as beam collision or numerical instability due to excessive element deformation.

We selected four metamaterials from our sub‐library of 26 for fabrication (Figure S4, Supporting Information) and, ultimately, one metamaterial design for mechanical characterization. This selection was based on the computed material properties and our success in printing and post‐processing. We observed corresponding mechanical properties between the calculated and measured stiffness for the X‐axis (14.1 kPa vs 23.6 kPa) and the Z‐axis (41.2 kPa vs 43.3 kPa), but not for the Y‐axis (45.4 kPa vs 6.9 kPa) (Figure 2C). Additionally, while compressing the metamaterial, we noted an auxetic response (Figure 2D). The variance between the computational and experimental results is likely due to discrepancies between the metamaterial design and the printed structure (Figure 2E). For instance, certain beams did not print correctly (warped or broken), while others were over‐polymerized (Figure S5, Supporting Information).

### 2.3 Cardiac Tissue Engineering using 3D Printed PCL Scaffolds

**Figure 3:**
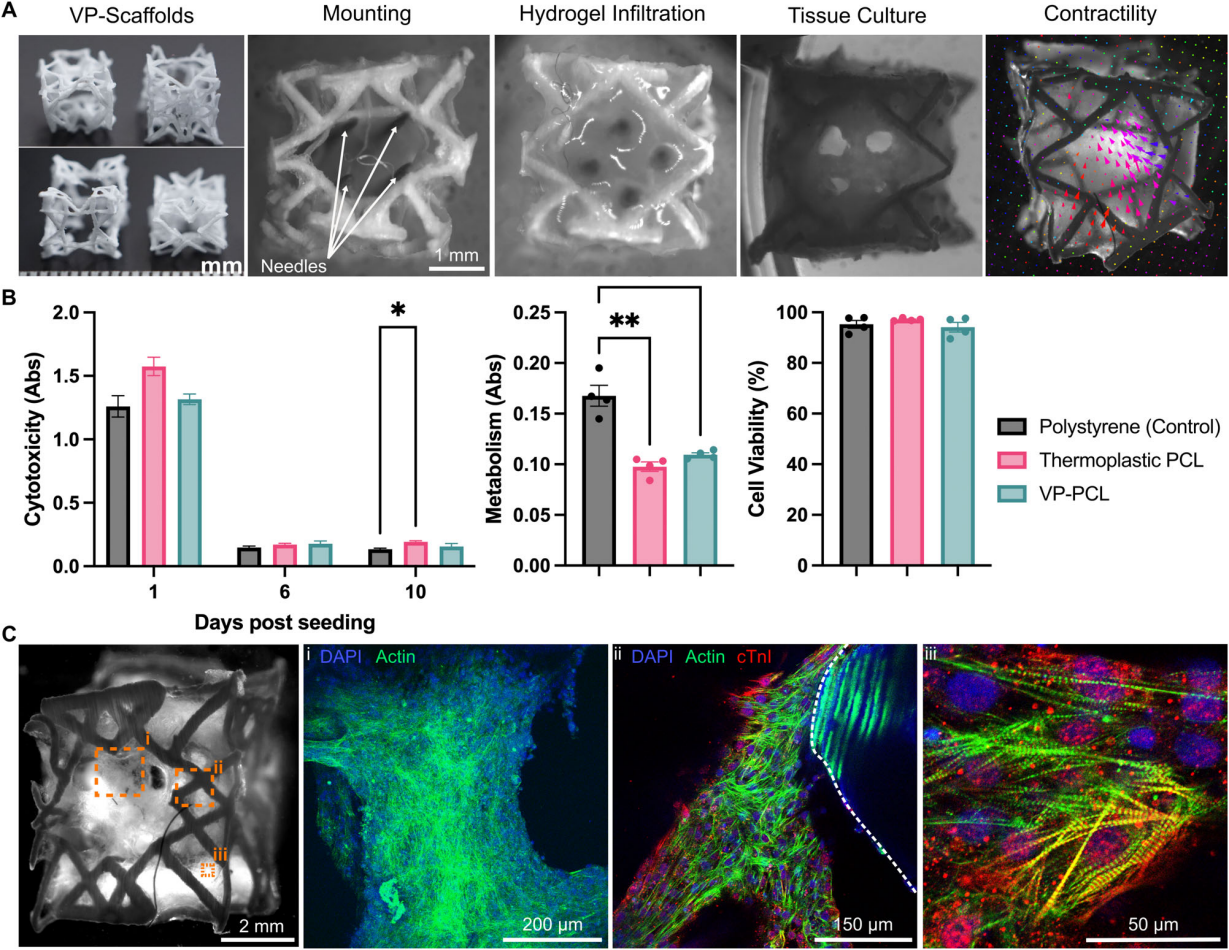
Biofabrication, Biocompatibility, and Structural Characterization of Reinforced Cardiac Tissues. A) Biofabrication of reinforced cardiac tissues. Cardiac metamaterials are infiltrated with cardiomyocytes in a fibrin hydrogel and cultured, resulting in contractile cardiac tissues. B) Biocompatibility assays of cardiomyocytes on different polymer substrates: cytotoxicity/LDH assay (left) (*n* = 5, two‐way ANOVA), metabolism/MTT assay (middle) (*n* = 4, one‐way ANOVA) and cell viability (right) (*n* = 4, one‐way ANOVA, ^*^ *p* < 0.05 and ^**^ *p* < 0.01). C) Immunofluorescence staining of cardiomyocytes in infiltrated metamaterials. Panel 1 is a maximum intensity Z‐projection (330 µm with 5 µm spacing). In panel ii, a dotted white line annotation highlights the separation between the PCL and cardiac tissue/cells.

Our next objective was to engineer reinforced cardiac tissues using our 3D‐printed metamaterial scaffolds (**Figure** **3**). We infiltrated PCL metamaterials with cardiomyocytes (100 × 10^6^ cells mL^−1^) in a fibrin hydrogel, which was injected into the scaffold (Figure 3A; Figure S6, Supporting Information). To ensure consistent and well‐distributed infiltration, we inserted needles into the central void of the metamaterial. This approach provides additional wetting surfaces, enhancing infiltration and stabilizing tissue formation during compaction and contraction (Figure S7, Supporting Information). Additionally, the channels may enhance tissue perfusion, and reduce cell hypoxia (Figure S8, Supporting Information). The resulting EHTs were contractile within ≈ 3 days and predominantly contracted along the softer metamaterial axis (Video S1, Supporting Information).

We performed an in‐depth analysis of cytocompatibility, metabolism, and viability to evaluate the compatibility of cardiomyocytes with VP‐PCL (Figure 3B). Specifically, we assessed cytotoxicity, metabolism, and cell viability by seeding cells in hydrogel on various polymer substrates. Cells seeded on VP‐PCL were compared to thermoplastic PCL and polystyrene (tissue culture plastic) controls. Our cytotoxicity assay revealed no significant differences among VP‐PCL, thermoplastic PCL, and polystyrene across days 1, 6, and 10. Cell metabolism was lower on both PCL substrates than on polystyrene, but cell viability remained comparable across all polymer substrates. These findings suggest that VP‐PCL has a similar cytocompatibility profile for cardiomyocytes to polystyrene, a widely used substrate for cell culture.

We performed immunofluorescence staining to evaluate the morphology of cardiomyocytes and EHTs (Figure 3C). Our EHTs contain bundles of aligned cardiomyocytes that adhere to the PCL metamaterial at multiple locations. The cardiomyocytes appear healthy and elongated, with localized sarcomere alignment influenced by local mechanical strain. Some cells exhibit sarcomere striation, confirming their structural organization. However, no global alignment direction was observed, as no external alignment methodology was applied.

### 2.4 Using Reinforced Cardiac Metamaterials for Implantation

**Figure 4:**
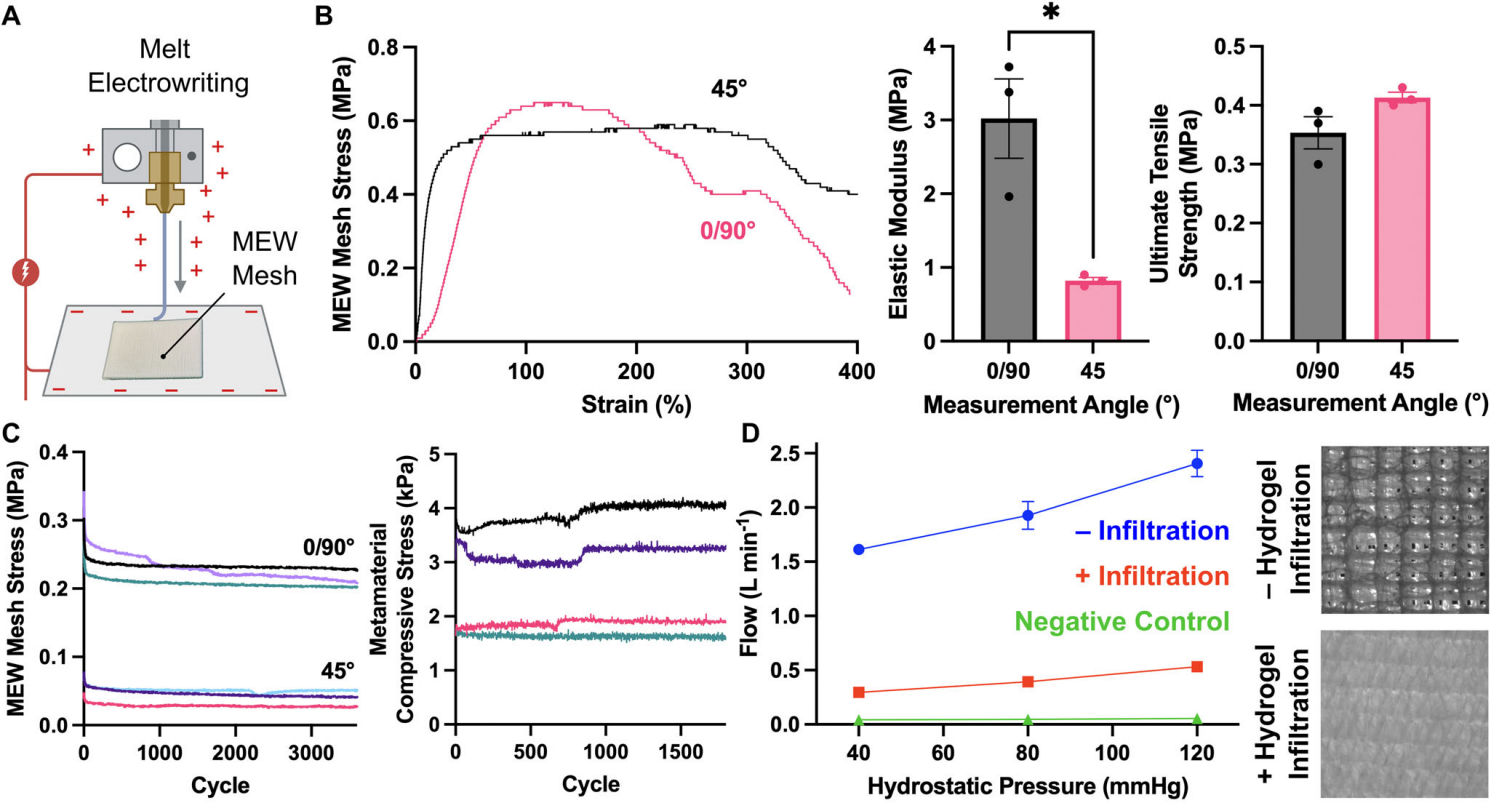
Mechanical Properties, Durability, and Permeability of the RCPatch. A) Schematic showing mesh fabrication using melt‐electrowriting (MEW). B) Mechanical characterization of MEW mesh (extension) (*n* = 3, unpaired *t*‐test, ^*^ *p* < 0.05). C) Cyclic loading of MEW mesh (15%, 3600 cycles, extension, *n* = 3) and metamaterial (Y‐axis, 15%, 1800 cycles, compression, *n* = 4). D) In vitro flow measurement of MEW mesh with and without fibrin hydrogel infiltration (*n* = 3).

To successfully implant the RCPatch into a ventricular defect, we needed to overcome two key challenges: reducing the permeability of the metamaterial to prevent bleeding and ensuring the patch could be securely sutured to the myocardium. We integrated a melt‐electrowritten (MEW) mesh (**Figure** **4**) with the VP metamaterial scaffold to address these challenges. This was achieved by using a small amount of the photoink mixture to crosslink the MEW mesh to the VP metamaterial. MEW uses an electrically charged nozzle to deposit molten PCL fibers precisely, allowing the fabrication of highly controlled micro‐ and nanoscale structures (Figure 4A).^[^ ^44^, ^45^, ^46^ ^]^ We found that MEW meshes with a 0° to 90° grid pattern and a 1.65 mm thickness (± 0.009 mm, SEM, *n* = 3) maintain deformability within a considerable elastic range under expansion (Figure 4B).

We assessed the cyclic durability of the individual RCPatch components under 15% strain (Figure 4C). We tested the MEW mesh under extension (3600 cycles) and the metamaterial under compression (1800 cycles). Both components exhibited stable mechanical behavior during repeated extension and compression. The MEW mesh showed minor yielding within the first 100 cycles, followed by consistent peak stress values. The metamaterial shows a similar, consistent response. The tissue within the metamaterial also withstood the cyclic loading (see Video S2, Supporting Information for a time‐lapse video). Furthermore, we did not observe any noticeable changes in cell viability following the cyclic compression (Figure S9, Supporting Information). Together, these results indicate that the RCPatch can withstand dynamic mechanical loading without structural failure under sub‐failure cyclic loading.

We infiltrated the MEW scaffold with a fibrin hydrogel to reduce the permeability (Figure 4D). We tested the result of this infiltration on an in vitro flow setup. This experiment demonstrated that fluid flow through the mesh was reduced ≈ fivefold (from 2.4 to 0.5 L min^−1^) with hydrogel infiltration.

We prepared for a large animal (porcine) study to assess whether our RCPatch could be sutured, withstand heart contraction and blood pressure, and restore hemodynamic stability under physiological conditions. We first performed an ex vivo implantation experiment to test the patch's handling, suturing, and the fitting of the metamaterial into the defect (Figure S10, Supporting Information).

**Figure 5:**
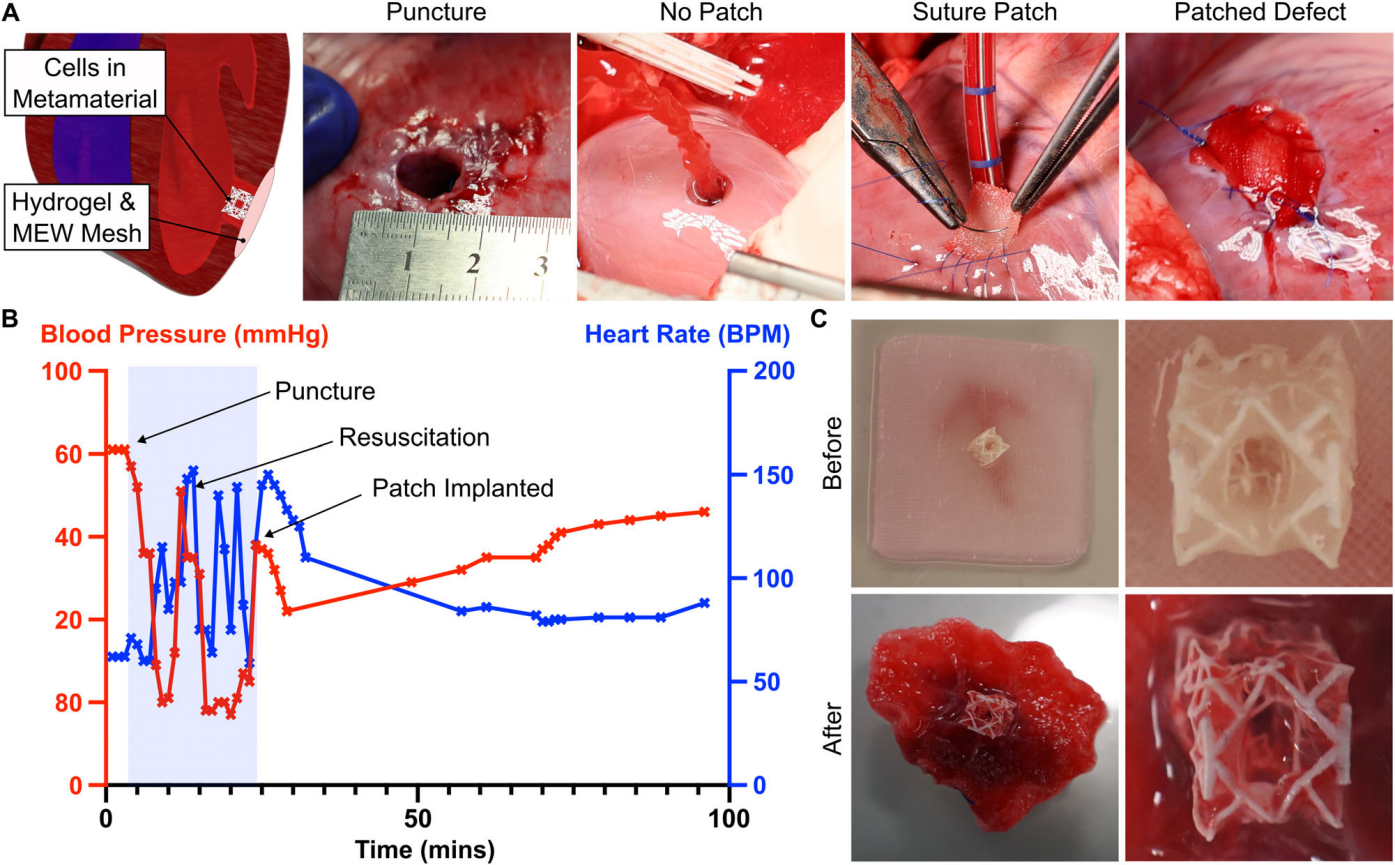
Acute Large Animal Trial using the RCPatch. A) The RCPatch consists of an MEW scaffold combined with cardiomyocytes in a VP Metamaterial. The patch was implanted near the heart's apex and exposed to left ventricular pressure (80 mmHg). B) Overview of animal condition (blood pressure and heart rate) during patch implantation. The implantation procedure is highlighted in light blue. C) Images of the RCPatch before and after implantation. Note that the patch was cut to size for the implantation.

During the animal experiment, we generated a myocardial defect model by creating a hole (Ø8 mm) in the left ventricle near the apex of the heart (**Figure** **5**). Consequently, the patch must withstand the blood pressure in the left ventricle (≈ 80 mmHg). The RCPatch was implanted onto the cardiac epicardium, with the metamaterial filling the myocardial defect (Figure 5A; Video S3, Supporting Information for Surgery Overview).

Upon implantation, most bleeding from the defect ceased immediately. Minor bleeding was observed for ≈ 10 min before stopping entirely. We observed partial hemodynamic restabilization over the next 60 min, with the animal's blood pressure recovering to 66 mmHg, compared to 80 mmHg at baseline (Figure 5B; see Table S4 (Supporting Information) systolic/diastolic pressure). The experiment lasted ≈ 90 min, during which the RCPatch was implanted for ≈ 60 min.

After termination, the patch was explanted and examined (Figure 5C). The metamaterial structure remained intact, and hydrogel and cells were observed within the metamaterial (Figure S11, Supporting Information). We performed flow cytometry to analyze the cells within the patch. We observed a large amount of red blood cell infiltration within the patch, as well as other cells from the animal (Figure S12, Supporting Information).

## 3 Discussion

In this work, we developed an intraventricular, reinforced cardiac patch (RCPatch) by combining a cardiomyocyte‐infiltrated, volumetrically 3D‐printed (VP) metamaterial and a melt‐electrowritten (MEW) mesh. This approach integrates the properties of stiff, tunable materials with soft, cell‐laden hydrogels. The metamaterial was designed by computational modeling and experimental validation, resulting in an anisotropic structure with mechanical properties that match those of the myocardium. The metamaterial is infiltrated with a cardiomyocyte‐laden hydrogel to form a reinforced cardiac tissue. To reduce the permeability of the patch, and enable implantation via suturing, we added the hydrogel‐infiltrated MEW mesh. When implanted into an acute porcine model with an induced myocardial defect, the RCPatch withstood ≈ 80 mmHg, minimized bleeding, and enabled partial hemodynamic restabilization. Our work advances cardiac patch design by moving beyond traditional surface patches toward 3D, implantable, mechanically stable tissues that can replace parts of the ventricular wall.

The current surgical state‐of‐the‐art materials for cardiac tissue repair (BPPs) provide mechanical support but suffer from calcification, thrombosis, and inflammation, and are unsuitable for tissue engineering.^[^ ^9^, ^10^, ^11^, ^12^, ^13^ ^]^ Alternative materials, such as EHTs and hydrogels, offer a regenerative option but lack the mechanical strength to withstand suturing, blood pressure, and intraventricular contraction.^[^ ^8^, ^20^, ^31^, ^32^, ^33^, ^34^ ^]^ Here, we used truss‐based metamaterials as a porous scaffold that could be infiltrated with cells, resulting in reinforced cardiac tissues. Furthermore, by utilizing metamaterials, we can tune our material properties to match those of the target tissue, which may enhance cell functionality and integration with the native tissue.^[^ ^39^, ^40^, ^47^ ^]^

To design our materials, we combined graph‐based generative modeling and finite element analysis to establish a scalable, data‐driven framework for inverse design. Traditional biomaterial design approaches often rely on manual trial‐and‐error or limited parametric sweeps, which constrain the ability to explore complex architectures and optimize mechanical behavior.^[^ ^20^, ^40^ ^]^ In contrast, our pipeline enables the tailored creation of architected bio‐metamaterials with anisotropic and tunable mechanical properties. This is also the first application of graph‐based inverse design to engineer metamaterials specifically for cardiac repair.^[^ ^48^ ^]^

The combination of VP and MEW techniques overcomes the individual limitations of each method. VP with PCL^[^ ^35^ ^]^ enables the fabrication of complex, high‐resolution scaffolds with tunable mechanical properties, while the MEW mesh provides a sutureable and impermeable layer, which is critical for intraventricular applications. The woven and flexible nature of the MEW mesh is similar to that of surgical materials, such as polyester (Dacron) and polypropylene meshes.^[^ ^7^, ^46^ ^]^ Furthermore, the ability to print larger MEW meshes (up to 50 mm × 50 mm, determined by collector size) allows surgeons to cut the materials to size based on the defect geometry. Finally, a key success factor in the RCPatch implantability is the MEW mesh's ability to withstand pressure and the dynamic expansion/compression of the heart.^[^ ^49^, ^50^, ^51^ ^]^ In the future, the geometry of the MEW mesh could be tuned to match the properties of the myocardium, such as auxeticity.^[^ ^49^ ^]^ Additionally, an integrated printing technology that combines MEW and VP could expedite patch fabrication.^[^ ^52^ ^]^

Some challenges remain: VP is currently limited in its scalability for fabricating larger metamaterials; this restricted us to fabricating single‐unit‐cell metamaterials. The resulting materials may lack the emergent behaviors that occur in larger metamaterial arrays, and withstand only mm‐scale deformations.^[^ ^43^ ^]^ This size limitation could be addressed by optimizing the resin chemistry to minimize light attenuation^[^ ^53^ ^]^, developing methods to increase the printable volume^[^ ^54^ ^]^ or using alternative 3D printing techniques.^[^ ^55^, ^56^ ^]^ Critically, enlarging the metamaterial would enable better surgical handling, improve mechanical robustness, and enhance regenerative potential. This approach should be used in conjunction with complementary tissue maturation (e.g., mechanical stimulation) methods to enhance overall tissue robustness.

The limited size of the metamaterial also led us to induce a small myocardial defect (Ø8 mm), whereas clinical myocardial injuries are often larger and irregular. To enable effective fitting, modular metamaterials could be fabricated to match the patient's specific anatomy. Additionally, sealing of the patch‐tissue interface may have been facilitated by clot formation, which could promote integration and should be further investigated.

Several future studies are required for the clinical translation of this research. First, the study was designed as an acute, proof‐of‐concept investigation using an animal model (*n* = 1) to demonstrate the basic function of the RCPatch. We demonstrated key functional parameters (suturability, mechanical stability, and prevention of bleeding); however, the patch's efficacy in a chronic setting must be established. Long‐term studies should assess cell survival and retention, vascularization, biodegradation, calcification, thrombosis, and functional recovery (e.g., improved ejection fraction) in animal infarct models.

Next, a dedicated electrophysiological evaluation will be essential to quantify action potential propagation, assess the electrical coupling between the graft and host tissue, and evaluate the potential for arrhythmogenicity following implantation. Despite this limitation, we observed synchronous contraction of the tissue in vitro and no arrhythmia in vivo. Lastly, while the patch was developed in reference to bovine pericardial patches, no direct comparisons were made. Further comparative studies in larger cohorts are needed to rigorously assess the long‐term functional and regenerative benefits.

In summary, our study presents a versatile approach for designing implantable 3D cardiac tissue patches with tunable mechanical properties. Using VP, we fabricated PCL metamaterials that support cardiomyocyte infiltration. Integrating these metamaterials with hydrogel‐infiltrated MEW meshes enabled suturing, reduced permeability, and prevented bleeding during implantation. In an acute large animal model, the RCPatch withstood intraventricular pressure and enabled partial hemodynamic restabilization. This approach provides a foundation for advancing the design of 3D, implantable, mechanically robust, and biological cardiac tissue patches, marking an important step toward translating engineered cardiac tissues into clinical practice for myocardial defect repair.

## 4 Experimental Section

### Metamaterial Design and Evaluation

Truss metamaterial designs were generated using a graph‐based inverse design framework^[^ ^39^ ^]^ built on a variational autoencoder (VAE) trained to encode over 900 000 truss architectures and their stiffness properties into a continuous latent space. A total of 500 initial latent vectors were selected from the training dataset, to balance computational efficiency and design diversity, and optimized using gradient descent. 327 optimized architectures were selected based on FE validation and manufacturability constraints for further study. Metamaterial candidates were evaluated using finite element (FE) simulations in COMSOL Multiphysics, employing a linear elastic material model with geometric nonlinearities. Using this setup, the equilibrium configuration of each single unit cell architecture was computed under a prescribed strain. No contact model was implemented to maximize computational efficiency. Further details on metamaterial design can be found in Text S1 (Supporting Information).

### Volumetric 3D Printing of Metamaterials

A photocurable PCL (VP‐PCL) was prepared as previously described.^[^ ^35^ ^]^ The resin mixture contained PCL (1 g, 346 µmol), diphenyl(2,4,6‐trimethylbenzoyl)phosphine oxide (TPO) (0.816 mg, 2.34 µmol), TEMPO (0.0918 mg, 0.589 µmol), and pentaerythritol tetrakis(3‐mercaptopropionate) (32 µL, 85 µmol) in chloroform (214 µL). The thiol crosslinker was calculated to achieve a 1:1 thiol‐to‐ene ratio, for each batch of PCL. The PCL, TPO, and TEMPO mixture was first sonicated and heated to 50 °C, after which the thiol was added. The mixture was then immediately added to a borosilicate glass vial (O.D Ø10 mm, 1 mm thickness) and used for printing in a Tomolite (Readily 3D) using a dose of ≈ 250 mJ cm^−2^, and the maximum absorption coefficient (2 cm^−1^). After printing, the mixtures were solidified by cooling, removed from the vial, washed once in chloroform, several times in acetone, and finally precipitated in ethanol and water.

### Fabrication of Melt Electrowritten (MEW) Meshes

MEW meshes were fabricated using a custom‐built MEW device, using a nozzle‐to‐collector distance of 3.3 mm, and a polymer feed pressure of 1 bar. PCL was melted at 80 °C and extruded into a 0–90° grid pattern. These parameters were chosen based on established MEW protocols to ensure high‐fidelity fiber deposition and stable jet formation. In particular, the selected nozzle‐to‐collector distance minimized jet instability and improved fiber placement accuracy, while the applied feed pressure ensured consistent fiber diameter within the desired range.^[^ ^44^, ^46^ ^]^

The resulting scaffold had a pore size of 250 µm (wall spacing). The thickness of the scaffolds was measured using a 3D optical profilometer, before mechanical characterization. In preparation for the animal surgery, the VP metamaterial was attached to the MEW mesh by crosslinking the two structures together using the same formulation used in VP, and a UV torch.

### Cell Culture and Hydrogel Infiltration

All materials were purchased from Gibco unless otherwise stated. Human iPSCs were cultured according to a previously published procedure.^[^ ^57^ ^]^ Specifically, cells were cultured on iMatrix‐511 (Takara) coated plates in StemBrew medium (Miltenyi Biotec). The culture medium was changed every day. At 80% confluence, the iPSCs were seeded for cardiomyocyte differentiation following the instructions for the StemMACS CardioDiff Kit XF (Miltenyi Biotec). iPSCs were cultured for 24 h in mesoderm induction media to initiate the differentiation. Then, cells were cultured for an additional 24 h in cardiac maintenance and, afterward, cardiac induction media (24–48 h media change). Cardiomyocytes started to beat around day 7. At day 10, cells were detached using a 1:1 trypsin and Stempro accutase mixture, followed by collagenase diluted in RPME 1640 for cell dissociation.

A cell‐laden fibrin‐based hydrogel was prepared by mixing fibrinogen (2 mg mL^−1^), Matrigel (10% v/v), thrombin (50 U mL^−1^), and iPSC‐CMs (≈ 100 million cells mL^−1^) according to a previously defined protocol.^[^ ^58^ ^]^ The PCL scaffold was incubated in fibronectin (20 µg mL^−1^) for 30 min at 37 °C, and mounted upon minuten pins (2 × 2, 0.2 mm, Ento Sphinx) before hydrogel infiltration (see Text S3, Supporting Information for details). After infiltration, constructs were cultured in RPMI 1640 supplemented with B27 (2% v/v), FBS (10% v/v), Rock Inhibitor (2 µm) (StemMACS Y27632, Miltenyi Biotec), and Primocin (0.2% v/v) for 2–3 days and subsequently without the FBS and Rock Inhibitor. All cells were incubated at 37 °C in an atmosphere with 95% humidity and 5% CO_2_.

### Mechanical Testing

The mechanical properties of VP‐PCL were measured using an Instron ElectroPuls system with a 2527 Series DynaCell load cell (±1 kN). PCL dog bones were prepared (14 mm × 5.7 mm × 3 mm) by casting PCL into a mold and irradiating with UV. The mechanical properties of VP metamaterials were tested using the same system, but using a ±250 N load cell. Compression tests were performed at a strain rate of 1 mm min^−1^ at 37 °C (temperature chamber). Young's modulus was calculated using the first 20% of the strain.

The mechanical properties of the MEW scaffolds were measured using a Zwick Roell Z005 testing machine equipped with a 5 kN load cell and a preload of 0.05 N. The test was conducted at a crosshead displacement rate of 0.1 mm s^−1^. The specimens were rectangular, conforming to the ASTM F3510 standard, with a gauge length of 20 mm and a width of 10 mm. The MEW scaffolds' elastic moduli were calculated per ASTM D638‐22. The thickness of the samples was measured using a 3D optical profilometer (Sensofar S neox, Sensofar).

The cyclic loading tests of the MEW patch were conducted using an Instron E10000 with a 1kN load cell over 3600 cycles (1 Hz, 15% strain), as previously performed.^[^ ^59^ ^]^ The cyclic loading of the metamaterial was performed using a micro‐scale tension‐compression test system (CellScale, MicroSquisher) over 1800 cycles (≈ 0.2 Hz, 15% strain).

### Optical Flow Characterization

Optical flow analysis was conducted, as previously performed, using an open‐source (OpenCV) optical flow algorithm to visualize tissue contraction.^[^ ^60^ ^]^ The contraction direction was colored using a hue, saturation, value (HSV) wheel. Arrow size and opacity were scaled with the magnitude of contraction. For visibility, arrow length was scaled by a factor of 25, and only a subset of arrows were shown by uniformly sampling 1% of the pixels.

### Fluid Permeability Testing

Hydrogel‐infiltrated MEW meshes were tested for permeability using an in vitro flow setup. The meshes were mounted in a hydrostatic water column, and flow was measured at 40, 80, and 120 mmHg over 30 s. A specialized insert was used to mount and expose the mesh (Ø6 mm) to the hydrostatic pressure.

### Cell Assays

Cells within a hydrogel were added to a 96‐well plate and polymerized. Different polymer discs (thermoplastic PCL (Facilan PCL 100 Filament), VP‐PCL) were then added on top of the cell/hydrogel mixture. The polystyrene control refers to tissue culture plastic, therefore no disc was added. The LDH assay (CyQUANT LDH Cytotoxicity Assay, Thermo Fisher) was used on the supernatant according to the manufacturer's instructions. The MTT Assay (CyQUANT MTT Cell Viability Assay, Thermo Fisher) was performed according to the manufacturer's instructions. An additional incubation step in Collagenase IV (5 mg mL^−1^) for 60 min was performed to release formazan crystals and ensure complete crystal dissolution. LDH and MTT Assays were evaluated on a plate reader (Tecan Infinite M200 Pro). The Live/Dead Assay (LIVE/DEAD Fixable Red Dead Cell Stain Kit, for 488 nm excitation) was used according to the manufacturer's instructions. Before performing the assay, the tissues were fully digested in Collagenase IV (5 mg mL^−1^) for 60 min. The assay results were evaluated using Flow Cytometry (LSRFortessa, BD Biosciences).

### Large Animal Study

Animal handling procedures were performed under the Canton of Zurich guidelines for animal experimentation (ZH072/2023). The metamaterial of the RCP patch was infiltrated with cells/hydrogel 5 days before the animal surgery. On the day of the surgery, the MEW mesh was infiltrated with a fibrinogen hydrogel (2 mg mL^−1^). The animal was anesthetized, intubated, and mechanically ventilated. A median sternotomy was performed to access the heart. A biopsy punch was used to create an Ø8 mm hole through the left ventricular wall near the apex of the heart. The RCPatch was trimmed and positioned over the defect, with the VP metamaterial placed inside the ventricular wall. The MEW mesh was sutured onto the epicardium using 4‐0 Polypropylene sutures. Blood pressure, ECG, and oxygen saturation were monitored continuously. Hemodynamic restabilization of the animal was assessed over 60 min, after which the animal was sacrificed, and the patch was explanted.

### Immunofluorescence and Microscopy

Optical microscopy was performed using an inverted microscope (Olympus CKX41, with cellSens) or a stereomicroscope (Zeiss Stemi 508, with pylon by Basler). Immunostaining was performed by first washing the tissues in PBS and fixing them in 4% paraformaldehyde for 30 min at 23 °C. The tissues were permeabilized with Triton‐X 100 (0.1%) in PBS for 30 min before blocking with donkey serum (5% v/v) in PBS for 2 h. Tissues were then incubated with primary antibodies (1/100, Anti‐Cardiac Troponin I antibody (ab47003) in donkey serum (5%) overnight at 4 °C. Next, the tissues were washed with Tween‐20 (0.1%, v/v), and incubated with secondary antibody (1/1000 Donkey Anti‐Rabbit IgG H&L in donkey serum (5%) at 24 °C, for 2 h. After which an actin stain (ActinGreen 488, ReadyProbes, Thermo Fisher Scientific) was added for a further 2 h. Cell nuclei were stained in DAPI (300 µm) for 30 min at 23 °C. Samples were washed in Tween‐20 (0.1%, v/v), and then imaged on a Leica SP8‐AOBS‐CARS Confocal Microscope.

### Statistical Analysis

Mechanical characterization data were preprocessed to exclude artifacts caused by sample slippage, ensuring accurate Young's modulus calculation. Data was plotted and analyzed using GraphPad Prism (x64, v. 10.4.1). Data are presented as mean ± SEM (standard error of the mean). A T‐test (unpaired, gaussian distribution, two‐tailed) was performed in Figure 2A,B, one‐way ANOVA (unpaired, gaussian distribution, using a Tukey test) was performed in Figure 2C,B (MTT and Live/Dead Assay), a Two‐way ANOVA (full model with interaction; unpaired design; Sidak's multiple comparisons; Geisser‐Greenhouse correction) was performed in Figure 3B (LDH Assay). For all tests, the alpha was set to 0.05. Differences between the two experimental groups were judged to have statistical significance at ^*^ *p* < 0.05, ^**^ *p* < 0.01, and ^***^ *p* < 0.005; groups with no significant difference are not indicated.

## Conflict of Interest

The authors declare no conflict of interest.

## Supporting information



Supporting Information

Supplemental Video 1

Supplemental Video 2

Supplemental Video 3

## Data Availability

The data that support the findings of this study are available from the corresponding author upon reasonable request.
