# Supplementary material for: Volumetric 3D Printing and Melt‐Electrowriting to Fabricate Implantable Reinforced Cardiac Tissue Patches
Source: Adv Mater. 2025 Aug 5;37(45):2504765. doi: 10.1002/adma.202504765 (PMC12617023; doi:10.1002/adma.202504765)
Supplement: Supplementary file 1 — Supporting Information [file ADMA-37-2504765-s001.pdf]

ADVANCED MATERIALS

Supporting Information

for *Adv. Mater.*, DOI 10.1002/adma.202504765

Volumetric 3D Printing and Melt-Electrowriting to Fabricate Implantable Reinforced Cardiac Tissue Patches

Lewis S. Jones, Hector Rodriguez Cetina Biefer, Manuel Mekkattu, Quinten Thijssen, Alessio Amicone, Anna Bock, Miriam Weisskopf, Dennis Zorndt, Debora Meier, Li Zheng, Melanie Generali, Robert K. Katzschmann and Omer Dzemali*

Volumetric 3D Printing and Melt-Electrowriting to Fabricate Implantable Reinforced Cardiac Tissue Patches

Lewis S. Jones[1], Hector Rodriguez Cetina Biefer[2,3], Manuel Mekkattu[1], Quinten Thijssen[4], Alessio Amicone[5], Anna Bock[1], Miriam Weisskopf[6], Dennis Zorndt[7], Debora Meier[7], Li Zheng[8], Melanie Generali[7], Robert K. Katzschmann[1†], Omer Dzemali[2,3†]*

[1]Soft Robotics Laboratory, ETH Zurich, Tannenstrasse 3, 8092 Zurich, Switzerland

[2]Department of Cardiac Surgery, University Hospital Zurich, Rämistrasse 100, 8091 Zurich, Switzerland

[3]Department of Cardiac Surgery, City Hospital Zurich—Triemli, Center for Experimental and Translational Cardiology (CTEC), University of Zurich, Zurich, Switzerland

[4]Polymer Chemistry and Biomaterials Group, Centre of Macromolecular Chemistry, Department of Organic and Macromolecular Chemistry, Ghent University, Krijgslaan 281 S4, Ghent, 9000 Belgium

[5]Institute for Biomechanics, ETH Zurich, Gloriastrasse 37-39, 8092 Zurich, Switzerland.

[6]Center for Preclinical Development, University Hospital Zurich, University of Zurich, Strickhofstrasse 40a, 8057 Zurich, Switzerland

[7]Institute for Regenerative Medicine (IREM), University of Zurich, 8952 Schlieren, Switzerland

[8]Mechanics & Materials Lab, Department of Mechanical and Process Engineering, ETH Zurich, 8092 Zurich, Switzerland

This document includes Tables S1-S4, and Figures S1-S12.

Other Supplementary Materials for this manuscript include:

- Video S1 – Metamaterial contractility
- Video S2 – Metamaterial compression time lapse
- Video S3 – Overview of RCPatch implantation

Table S1: Overview of the cardiac patch functional requirements, properties, results, and proposed novelty. References

Functional Requirement	Explanation	Our Approach	Property	Current Result	Novelty	Comparable References
**Mechanical**						
Extendable/Compressable	Stretch and compress with systole/diastole.	Reinforcing PCL scaffold designs are deformable >20%. Metamaterial stiffness designed to match myocardium stiffness.	The cardiac patch can withstand acute implantation without being destroyed.	The patch was implanted during an acute animal experiment . After explantation no holes in the MEW scaffold were observed. The VP Metamaterial was slightly compressed but not destroyed.	First exaple of a 3D metamaterial tailored to a cardiac application. First example of using a MEW-enabled cardiac patch during a preclinical setting.	1, 2
High Burst Strength	Withstand pressure generated during systole (~120 mmHg).	Hydrogel is reinforced with a fine MEW mesh.	Low initial leakage rate. Any leakage is quickly blocked by blood clotting.	There was minimal leakage in the patch after implantation. The animal regained hydrodynamic stability (80 mmHg blood pressure).	Demonstration of hemodynamic stability using MEW-enabled patch.	3
Tear Resistance	Withstands suturing and other local stress	Use of woven and flexible MEW mesh using high tensile strength material (PCL)	Patch can withstand suturing and does not tear leading to blood leakage.	The patch was implanted via suturing aalong the entire circumference of the patch. No breakage was observed.	One of the first examples of suturing a MEW mesh during a preclinical trial.	4
Fatigue Resistance	Withstands repetitive mechanical loading	Not investigated	N/A	N/A	N/A	3
**Chemical**						
Biodegradable	Ultimately degrades after recovery	Use of biodegradable materials (PCL)	Patch can support cardiac hemodynamic recovery and regeneration, and degrade over time.	Not investigated. However PCL is biodegradable.	N/A	5
Resistant to unfavourable biological processes (e.g., Calcification)	Material properties are not negatively affected by biological processes	Not investigated	N/A	Not investigated.	N/A	6
Enhances favourable biological processes	Supports cellular infiltration, adhesion, growth and promotes tissue recovery	Combine porous thermoplastic material with a biocompatible hydrogel; PCL is biocompatible.	Cardiomyocytes can be infiltrated into the patch; cells can adhere to patch.	Cardiomyocytes shown to be viable and functional when infiltrated into the VP Metamaterial.	Using 3D material demonstrates potential for large volume cardiac tissue engineering.	7,8
Conductive	Does not disrupt electrical signal propagation	Not investigated	N/A	N/A	N/A	
**Biological**						
Cytocompatibility	Can support cell growth and infiltration	Use cytocompatibility materials (PCL) and 3D printing procedures (MEW and VP).	Cells can be seeded/infiltrated into patch and show normal functionality.	Various cytocompatibility markers evaluated (Mitochondrial Metabolism, LDH release, viability). All results comparable to a control.	Demonstration of compatibility between VP-PCL and Cardiomyocytes.	6
Hemocompatible	Does not damage the blood	Not investigated	N/A	N/A	N/A	N/A
Biocompatible	Does not adversely affect biological tissues	Not investigated	N/A	N/A	N/A	6, 7
**Other**						
Customizable size and stiffness	Can be used for a range of defects.	3D printing approach (MEW) can fabricate large patches (25 cm2). Material properties of metamaterial are tuneable.	Large patches can be produced and cut to size as required. Specific stiffnesses can be selected according to tissue type/required properties.	Current MEW scaffold size overlaps with range of available commercial patch sizes. Metamaterial stiffness is tuneable within range of native myocardium stiffness.	Demonstration of tuneable stiffness for cardiac metamaterials show applicability of metamaterial-approach for tissue engineering large volume implants.	8
Handleable/Implantable	Can be handled by surgeons and implanted by the determined methodology.	Cells are protected within 3D printed metamaterial, MEW scaffold can support suturing and handling throughout surgery.	The cardiac patch can be implanted via standard surgical procedures.	The cardiac patch was successfully implanted.	Demonstration that MEW-scaffolds are well suited for suturing and other blood-contacting applications.	3

Table S2: Overview of possible 3D printing methods to fabricate cardiac metamaterials.

3D Printing Approach	Minimum Feature Size	Geometric Limitations	Material Limitations	Application Notes for Cardiac Patches
**Fused Deposition Modelling**	~400 µm	Overhangs require support, minimum feature size is material dependent	Uses thermoplastic materials	Materials tend to be biocompatible, but the difficulty in printing overhangs limits the 3D complexity of the parts.
**SLA/DLP**	~35-100 µm	Requires supports	Photopolymer resins	Can be used to produce detailed 3D parts, but very limited availability of cytocompatible resins.
**Multi-Photon Printing**	< 1 µm	Small print volume	Photopolymer resins	Print volume too small for cardiac patch application.
**Volumetric Printing**	~25 µm	Limited print size (cm3), no support structures required, post processing can limit feature size	Photopolymer resins	Aimed towards bioprint+F8ng, such as with hydrogels, so cytocompatible formulations available. Print size currently limited but can be overcome in the future. F5
**Powder Bed Fusion**	~ 100 µm	Thin features can warp, rough parts, no support structures required	Polymers, metals, and composites	Limited material selection.
**Vision Controlled Jetting**	10 µm	Post-processing can limit minimum feature sizes	Photopolymer resins	Can be used to produce complex 3D parts, but no availability of cytocompatible resins.

Table S3: Iteration of Photoink for Volumetric 3D Printing of PCL Metamaterials

Mixture	TPO:TEMPO Mol Ratio	TPO, TEMPO (mg)	Dose Range (mJ/cm²)	Outcome	Justification for Change
Original Mixture	1:0.67	1.632, 0.4896	1200–2500	Only printed at very high doses; no structure formed, or extreme blurring and no feature resolution.	High TEMPO inhibited radical polymerization; required very high doses.
Iteration 1 (1/2 TEMPO)	1:0.33	1.632, 0.2448	675–1000	Lower doses (≈750 mJ) achieved; structures non-homogenous (blurred and overpolymerized on edges), or underpolymerized and too fragile.	TEMPO reduction lowered required dose, but ideal printing window between overpolymerization and underpolymerization still very narrow.
Iteration 2 (1/2 TPO & TEMPO), absorption coefficient compensation)	1:0.33	0.816, 0.1224	800-1200	Prints significantly better; beams thinner and better resolved, but structures often warped or tore apart during post-processing.	Reducing TPO/TEMPO and performing absorption coefficient attenuation reduce light attenuation, improving crosslinking at center of vial. This optimization allowed us to make more homogenous structures, but post-processing remained an issue.
Iteration 3 (1/2 TEMPO), absorption coefficient compensation	1:0.17	0.816, 0.0612	250–275	Extremely sensitive to dose; structures overpolymerized (blurred features, fused beams); narrow printing window, minor dose changes (≈25 mJ) caused fragile prints or blurring.	Further TEMPO reduction enhanced crosslinking (allowing low dose printing) but sacrificed reproducibility.
Iteration 4 (slight increase in TEMPO), absorption coefficient compensation	1:0.25	0.816, 0.0918	300–400	Successful printing of 150-300 µm beams; structures were mechanically robust after washing; reduced overpolymerization; some minor heterogeneity between prints.	Slight TEMPO increase widening working dose range and improved reproducibility.

Table S4: Heart monitor parameters during patch implantation

Time (mins)	Stage	Blood Pressure (mmHg)			Heart Rate (BPM)
		Systolic	Diastolic	Mean	
**0-4**	Beginning	89-101	61-68	72-81	71-62
**4-24**	Implantation	25-68	12-47	17-56	152-60
**24-End**	Recovery	64-92	42-49	62-66	150-79

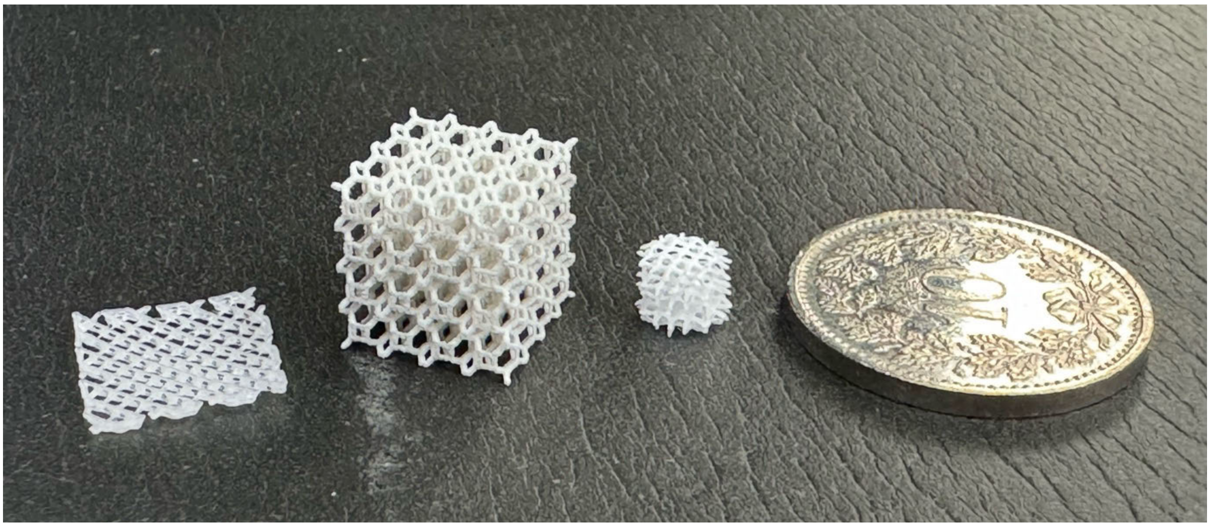

Figure S1: Attempted Metamaterial Manufacturing Processes. We investigated different manufacturing approaches before selecting Volumetric Printing (VP) as the preferred method. Left to right: FDM Printing, Vision Controlled Jetting (Inkbit), Volumetric Printing.

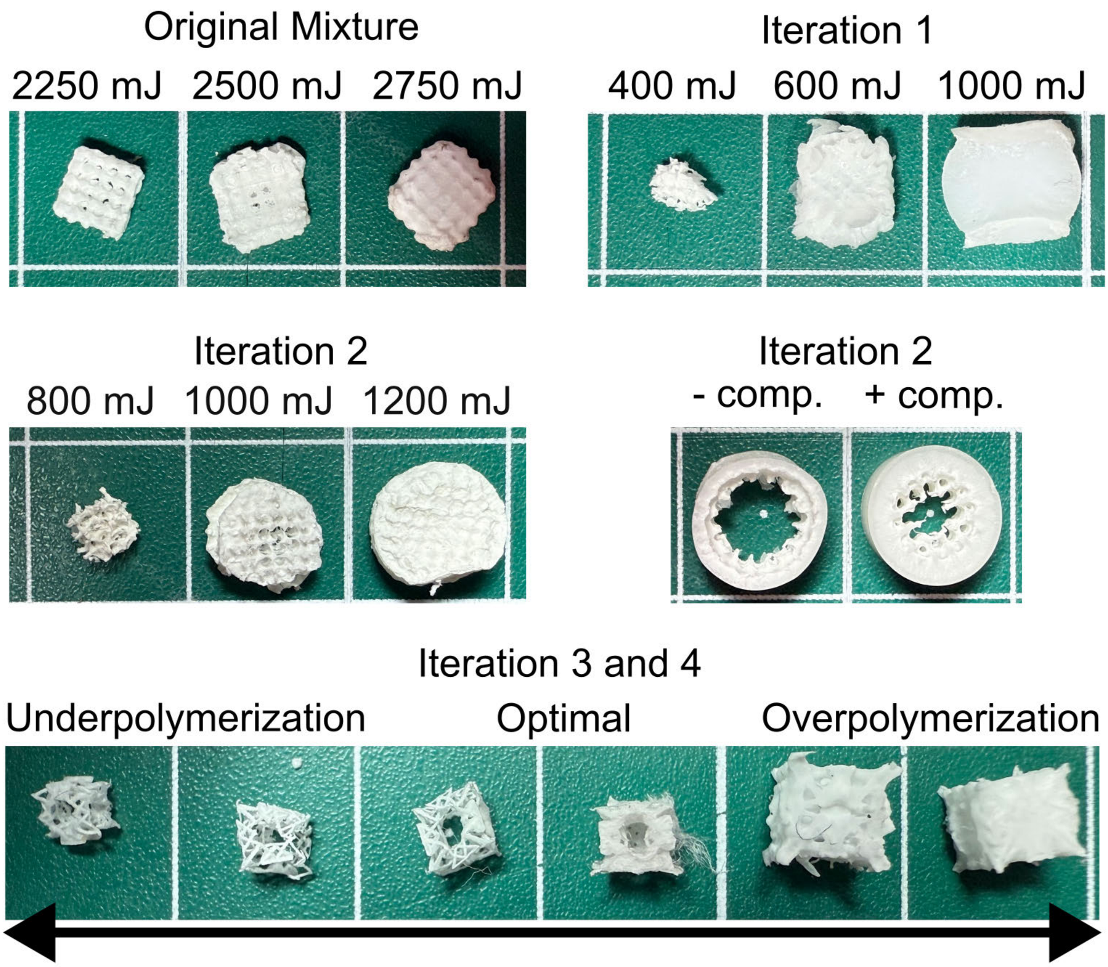

Figure S2: Iteration of Printing Parameters for Volumetric 3D Printing of Metamaterials. The corresponding chemical composition of the photoinks is presented in Table S3. Comp = compensation of absorption.

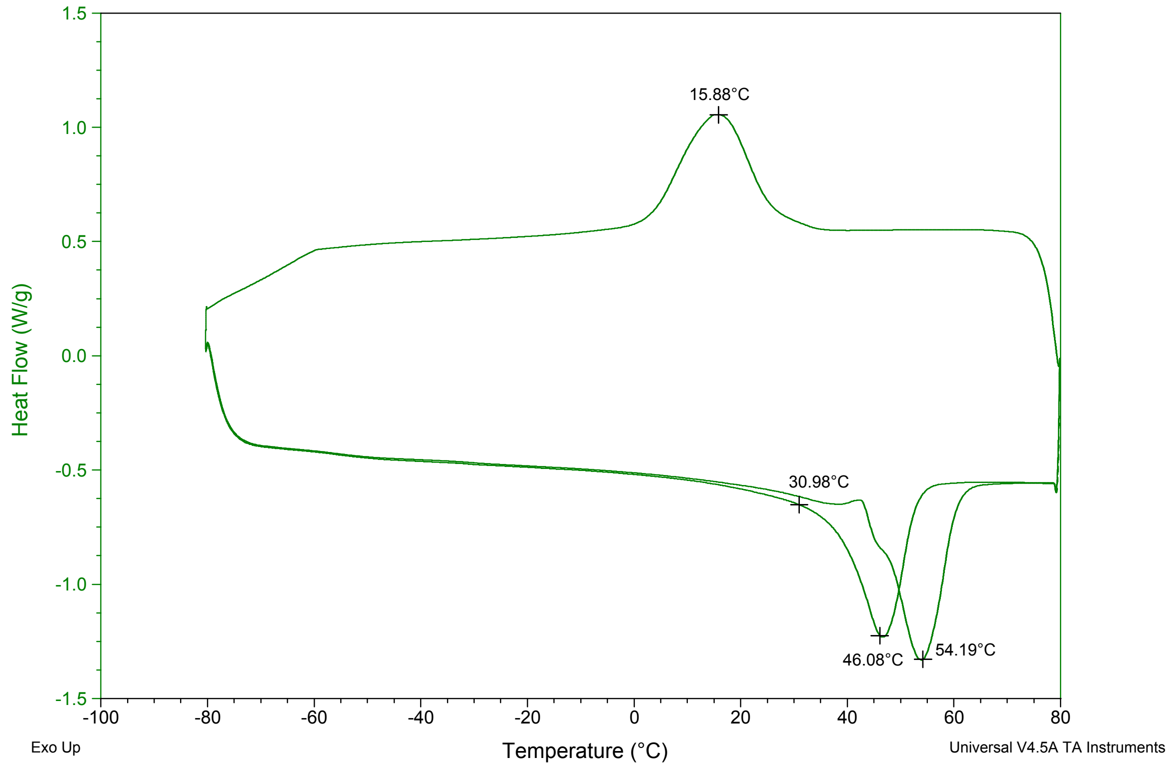

Figure S3: Differential scanning calorimetry of VP-PCL. The temperature transitions are labeled.

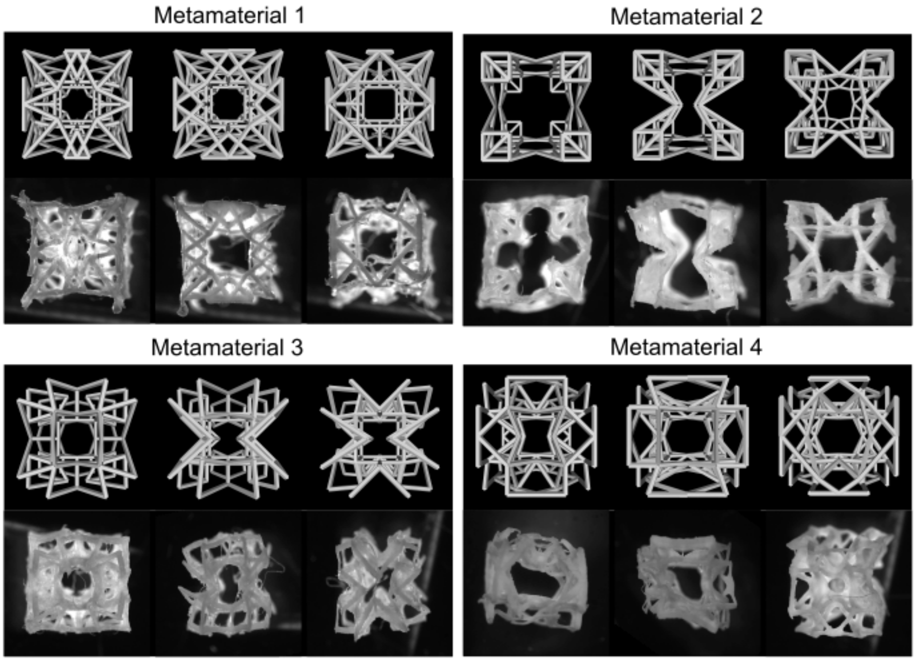

Figure S4: Metamaterial Model/Fabrication Comparison showing four selected metamaterial geometries.

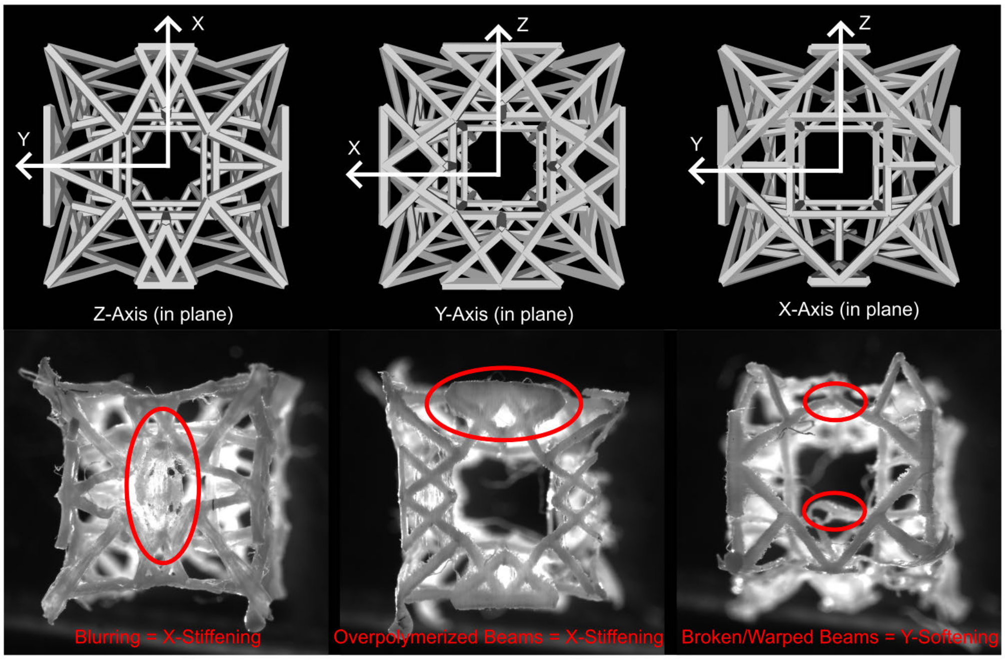

Figure S5: Annotated Metamaterial showing regions over polymerization, blurring, and beam warping/under polymerization.

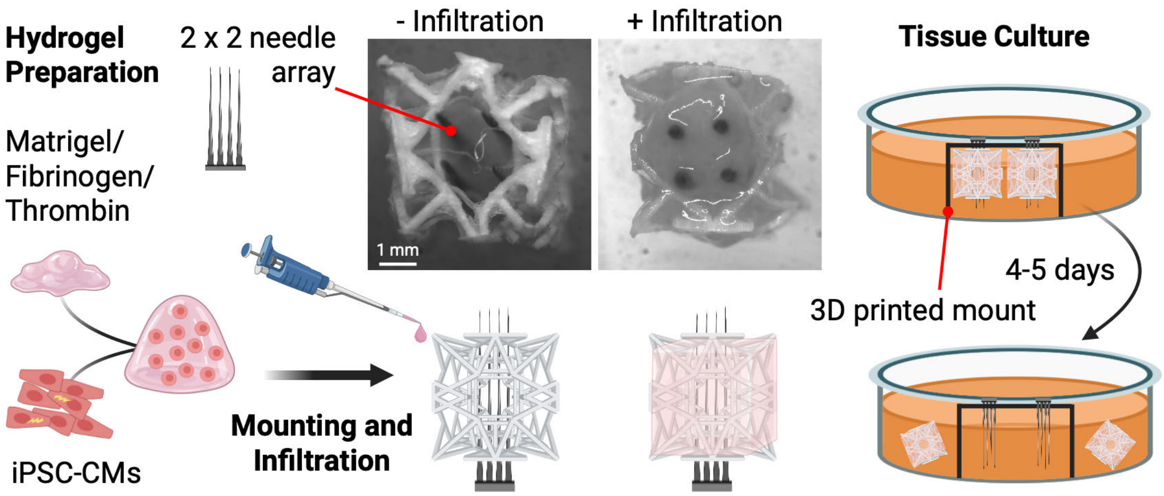

Figure S6: Overview of Metamaterial Infiltration. The corresponding information on infiltration can be found in Text S3.

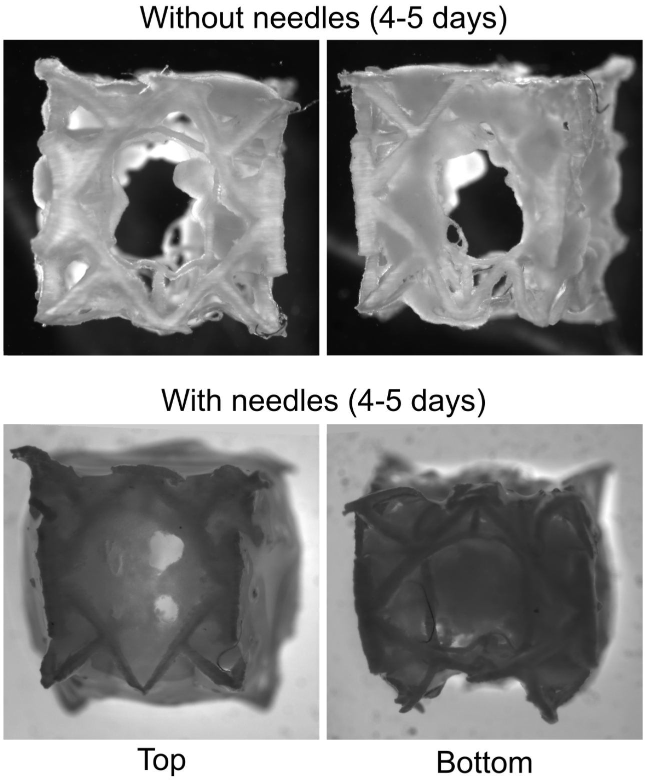

Figure S7: Comparison of Infiltrated Metamaterials, without (top) and with (bottom) needles.

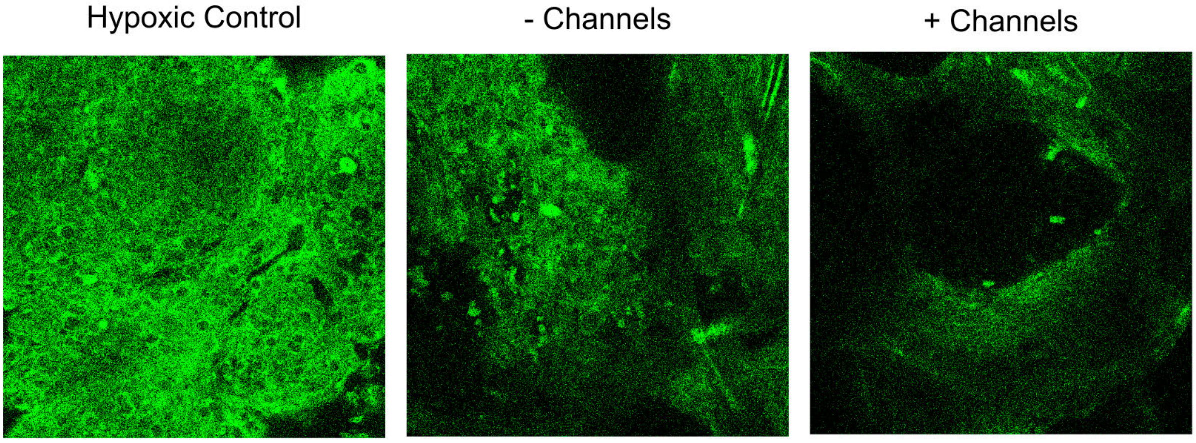

Figure S8: Hypoxia Assay in Tissues with and without channels. We used a fluorescently sensitive hypoxia assay to measure cell and tissue hypoxia in tissues with and without channels. We also performed a hypoxia control (cells in 100% CO_2 for 4 hours).

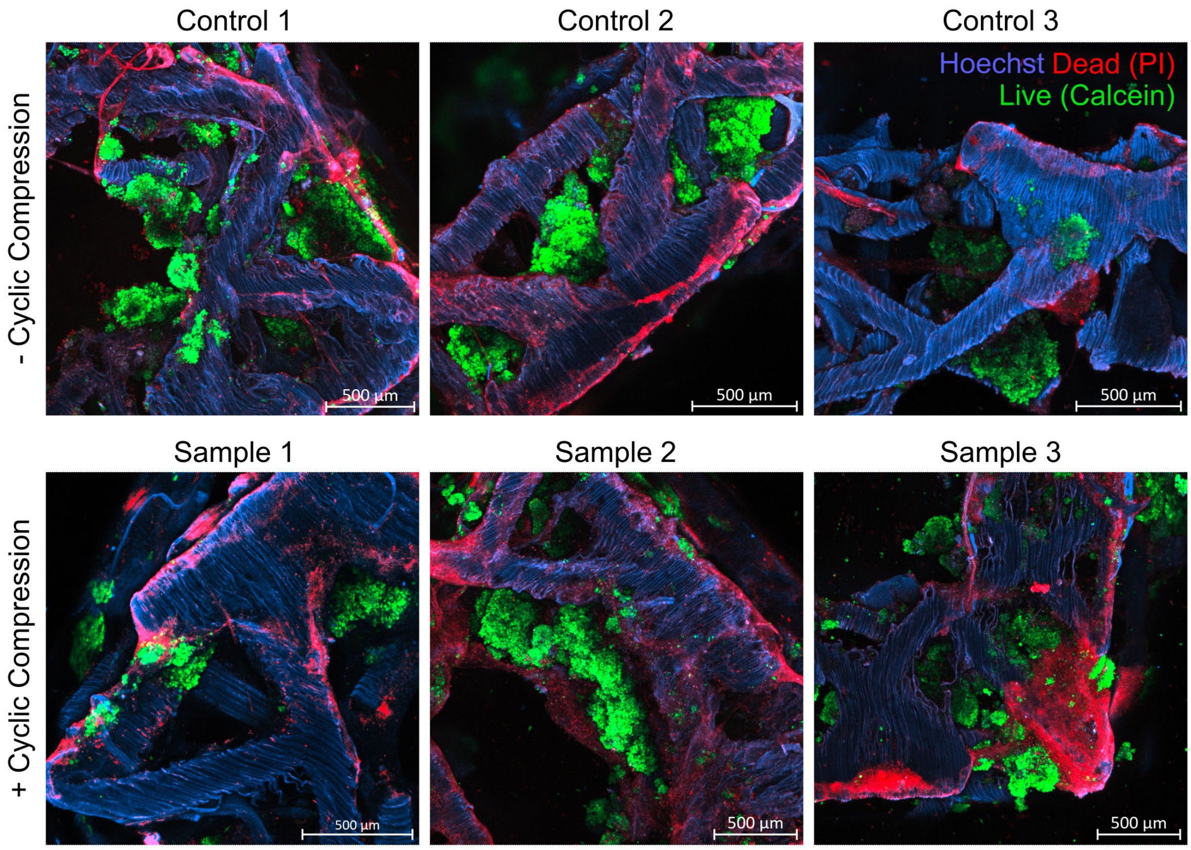

Figure S9: Live/Dead Assay (Immunofluorescence). Infiltrated scaffolds were subject to cyclic strain (15%, 1800 cycles), after which the cell viability was measured (images of the strain axis). PI = propidium iodide.

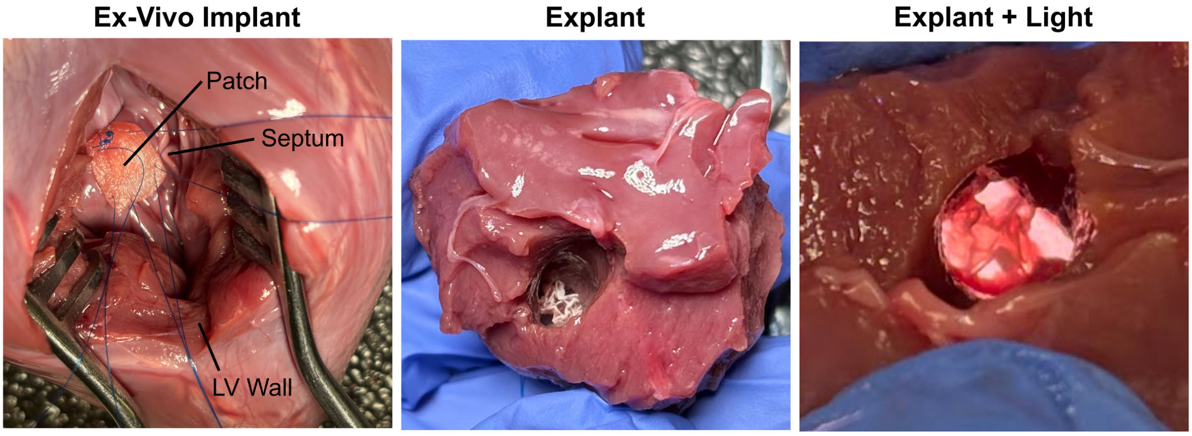

Figure S10: Fitting of RCPatch in Septum during ex-vivo experiments. Patch fitting was tested by puncturing a hole into the septum (⌀8 mm), and suturing the RCPatch onto the induced septal defect.

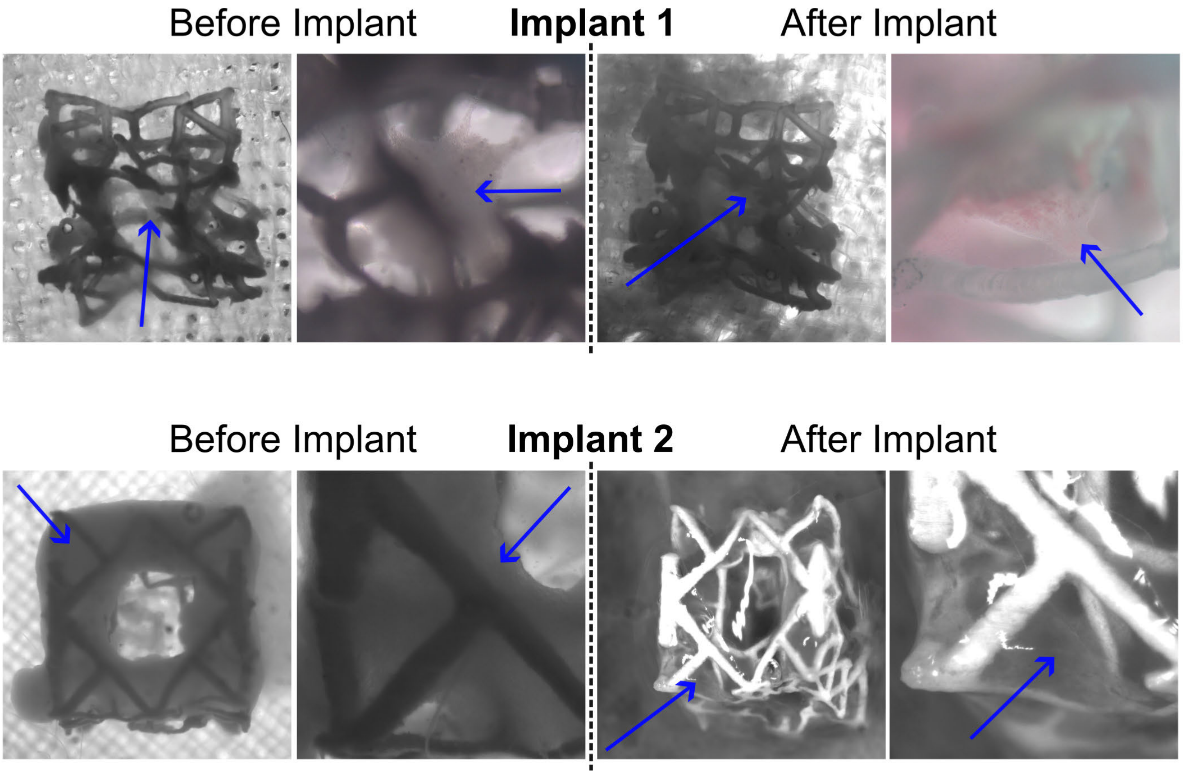

Figure S11: Comparison of RCPatch before and after implantation. The two different implants correspond to two different experiments. The blue arrows point to the engineered cardiac tissue contained within the metamaterial.

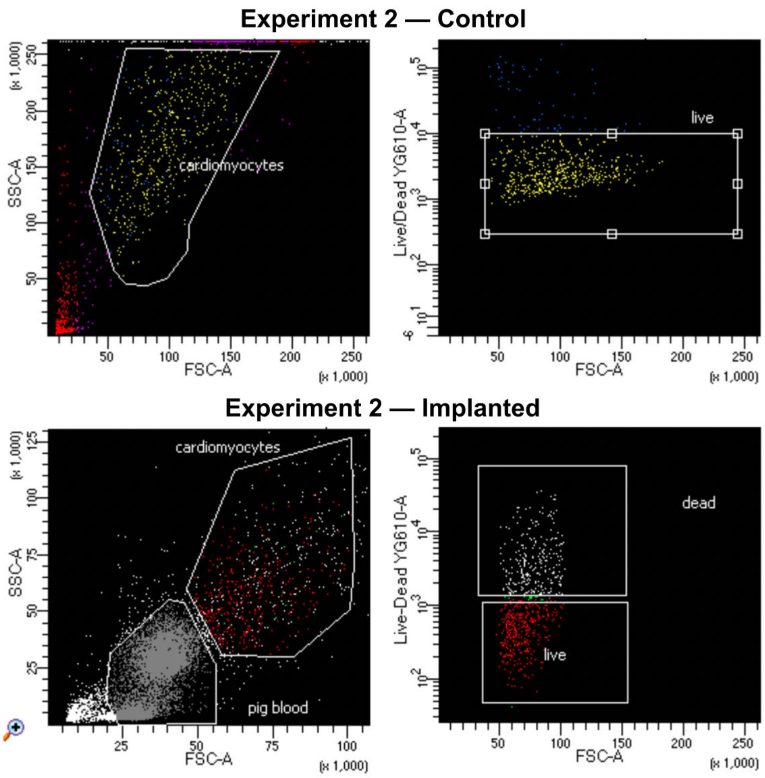

	Experiment 1		Experiment 2	
% of cells	Control	Implanted	Control	Implanted
Cells minus aggregates, debris and other cells	92.8	32.2	54.3	6
**Live (% target cells)**	91	91.5	88.6	53.8
**Dead (% target cells)**	8	2.4	11.4	43
**Excluded (% target cells)**	1	6.1	0	3.2

Figure S12: Flow Cytometry comparing cell viability after implantation. The flow cytometry data shows the analysis of the second (successful) implantation experiment.

Text S1: Background Information on the Inverse Design Framework

We used a graph-based inverse design framework to generate truss architectures with target mechanical properties. The framework is based on a variational autoencoder (VAE) jointly trained with a property prediction network using a dataset of 965,736 truss architectures. Each architecture is encoded as a graph representing its discrete geometry. The VAE maps these geometries into a continuous latent space, which captures geometry–property relationships in a compact, low-dimensional form. The property predictor estimates stiffness from the latent vectors. This setup enables gradient-based optimization toward target mechanical properties by providing differentiable access to structure–property relationships.

To identify truss architectures with desired stiffness, we evaluated all structures in the training dataset with respect to the target property. Specifically, we computed the distance between each architecture's predicted stiffness and the target stiffness using the trained property predictor. We then selected the 500 closest matches as initial guesses for optimization. This number reflects a trade-off between computational efficiency and design diversity; in the previous work, 100 initial guesses provided satisfactory results.

We optimized each of the 500 latent vectors independently using gradient descent, aiming to minimize the error between the predicted and target stiffness values. Optimization was performed in parallel for all candidates. After convergence, we decoded the optimized latent vectors into discrete truss architectures.

We validated each optimized architecture using finite element (FE) homogenization to assess the agreement between predicted and simulated stiffness values. The FE model used a linear elastic material law and incorporated geometric nonlinearities, which capture changes in geometry under large deformations. We computed the effective stiffness by solving for the equilibrium response under applied strain. No contact modeling was used to maintain computational efficiency.

We selected 327 from 500 optimized truss architectures based on two criteria. First, we validated the designs using finite element (FE) homogenization and retained only those whose FE-evaluated stiffness closely matched the target value. Second, we applied manufacturability constraints to exclude architectures with beam angles below 10° or with long horizontal members supported only at a single point, as such features are difficult to fabricate reliably.

Finally, the 327 metamaterials were evaluated as single unit cells using FE simulation (COMSOL Multiphysics with a linear elastic material model that incorporates geometric nonlinearities). This step was necessary to capture detailed deformation behavior, as the simulation setup used in the generative modeling framework (based on FE homogenization with periodic boundary conditions) does not account for localized effects relevant to fabrication and performance.

Text S2: VP-PCL Printing and Post-Processing Protocol and Optimization
The photoink formulation, printing parameters, and post-processing protocol were optimized to print lattice structures with beam diameters ranging from 200 to 300 µm. The photoink formulation was iterated to balance reactivity and attenuation by controlling the TEMPO:TPO ratio and concentration (Table S3). A recommended dose range for VP is between 300-800 mJ cm^{-2}, corresponding to a print time of under 30 seconds. Note that the printing parameters here are optimized for the specific vial diameter due to the attenuation of light (absorption ∝ path length).

The photoink was prepared in batches of 1-4 g and transferred to disposable borosilicate glass vials (outer diameter, 10 mm; thickness, 1 mm). Approximately three prints can be produced per gram of photo ink. To prepare the photoink, the PCL (1 g), TPO (0.816 mg, from a 40 mg/mL stock solution (in chloroform)), TEMPO (0.0918 mg, from a 4 mg/mL stock solution (in chloroform)), and chloroform was added and heated (50 °C)/sonicated/vortexed until mixed. Solutions of TPO and TEMPO are made fresh and used within a week. The PETA-4SH was added last, as it oxidizes. Printing should proceed immediately after adding the thiol. If thiol formation (cloudiness) is observed, degassing the mixture and avoiding oxygen is required. Note, heating the mixture accelerates thiol oxidation. Ideally, the mixture is kept warm to prevent recrystallization before printing. The mixture is light-sensitive.

During printing, the primary parameter optimized was dose. We recommend increasing the dose in 100 mJ cm^{-2} increments, identifying underpolymerization (where no structure forms and mechanically weak structures are present), and overpolymerization (where the structure blurs). Afterward, the dose can be optimized in smaller increments (25 mJ cm^{-2}). The only other

printing parameters to input are the absorption coefficient (2 cm^{-1}) and vial dimensions. All other settings were used as their default values.

To post-process the printed structures, the PCL is first reheated. A minimal volume of chloroform was added to submerge the print and dissolve the unreacted PCL with gentle agitation (the print is fragile). Ethanol was then layered on top of the chloroform, and the chloroform was removed using a syringe (inserted to the bottom of the vial). The scaffold was washed with ethanol multiple times to completely remove chloroform and any residual photoink and heated as needed to speed up dissolution. Finally, the print was recrystallized by slowly adding water and simultaneously swirling the print. During recrystallization, the scaffold becomes sticky and can adhere to the vial wall, causing warping of the print. The print was left in water overnight to recrystallize fully. Note that the mechanical integrity of the print is weakest in chloroform (most swollen) and ethanol. Therefore, the print must remain fully submerged in fluid. After precipitation in water (overnight or several hours), the print is mechanically stable.

Text S3: Further Details on Metamaterial Infiltration
For hydrogel preparation, a previously published protocol was followed. Metamaterials were infiltrated with a cell-laden hydrogel composed of Matrigel and fibrinogen. To support tissue formation within the central void of the metamaterial, an array of 2 × 2 microneedles (minuten pins, 200 μm diameter, 1 mm spacing) was used to suspend the scaffold during infiltration. This setup prevented contact with surrounding surfaces, thereby preserving surface tension and ensuring that the hydrogel remained confined within the scaffold structure. After infiltration, the constructs were incubated for 15 minutes to allow the hydrogel to fully cure before the addition of media. The scaffolds were then transferred to a custom 3D-printed stand within a well plate, designed to keep the metamaterials suspended above the plate bottom, reducing cell migration while minimizing the required media volume. Metamaterials either detached from the minuten pins spontaneously or were manually removed after a minimum of 5 days in culture.

References

[1] D. Olvera, M. Sohrabi Molina, G. Hendy, M. G. Monaghan, *Adv. Funct. Mater.* **2020**, *30*, 1909880.

[2] Z. Dong, X. Ren, B. Jia, X. Zhang, X. Wan, Y. Wu, H. Huang, *Mater. Today Bio* **2024**, *26*, 101098.

[3] A. S. Federici, O. Garcia, D. J. Kelly, D. A. Hoey, *Adv. Funct. Mater.* **2024**, *34*, 2409883.

[4] Y. Han, M. Lian, B. Sun, B. Jia, Q. Wu, Z. Qiao, K. Dai, *Theranostics* **2020**, *10*, 10214.

[5] Q. Thijssen, A. Quaak, J. Toombs, E. De Vlieghere, L. Parmentier, H. Taylor, S. Van Vlierberghe, *Adv. Mater.* **2023**, *35*, 2210136.

[6] P. R. Schmitt, K. D. Dwyer, K. L. K. Coulombe, *ACS Appl. Bio Mater.* **2022**, *5*, 2461.

[7] W. Y. Yeong, N. Sudarmadji, H. Y. Yu, C. K. Chua, K. F. Leong, S. S. Venkatraman, Y. C. F. Boey, L. P. Tan, *Acta Biomater.* **2010**, *6*, 2028.

[8] J. H. Park, H.-J. Park, S. J. Tucker, S. K. Rutledge, L. Wang, M. E. Davis, S. J. Hollister, *Adv. Funct. Mater.* **2023**, *33*, 2215220.
